# Menaquinone 7 Stability of Formulations and Its Relationship with Purity Profile

**DOI:** 10.3390/molecules24050829

**Published:** 2019-02-26

**Authors:** Patrick Orlando, Sonia Silvestri, Fabio Marcheggiani, Ilenia Cirilli, Luca Tiano

**Affiliations:** 1 Department of Life and Environmental Sciences, Polytechnic University of Marche, 60131 Ancona, Italy; p.orlando@univpm.it (P.O.); s.silvestri@univpm.it (S.S.); f.marcheggiani@univpm.it (F.M.); 2 Department of Clinical Sciences, Polytechnic University of Marche, 60131 Ancona, Italy; ilenia.cirilli@gmail.com

**Keywords:** MK7, stability, purity, food supplement

## Abstract

Menaquinone-7 (MK7) is a member of the vitamin K family in which interest has considerably increased over the last decade, mainly due to its beneficial role in human health. MK7 can be produced by synthesis or fermentation, and its purity profile can differ depending on methodologies and extraction procedures. Finished formulations show a high heterogeneity of purity profiles, as well as frequent discrepancies in the nominal content, compared to the actual title. The present study compared purity profiles of different raw material and related them to their stability in normal (12 months/25 °C/60%RH) and accelerated conditions (6 months/40 °C/75% RH) in order to test their performance in the presence of different common excipients. Results showed higher purity profile results in enhanced stability, and this could explain title discrepancies found in finished products, which are present on the market worldwide.

## 1. Introduction

**Figure 1:**
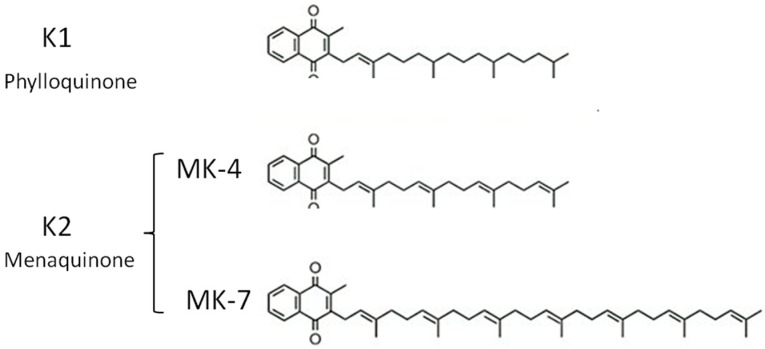
K structures. MK4 and MK7 are the major forms of K2 present in the diet.

Vitamin K constitutes a family of compounds with a common chemical structure, 2-methyl-1,4-napthoquinone (Figure 1). Despite their difference in molecular structure, the molecules of this family share a common function as a specific cofactor in the formation of γ-carboxyglutamyl (Gla) from specific glutamate residues in VKDP. The Gla residues confer calcium-binding properties; this common pattern characterizes different functions of vitamin K-dependent proteins (VKDP) [1,2,3,4,5]. The “vitamin K” family comprises three subtypes of molecules, K1, K2, and K3. Among them, vitamin K1 (phylloquinone), of plant origin, is the predominant dietary form of vitamin K [6,7,8]; the second main component of the vitamin K family is vitamin K2 (menaquinone), primarily of bacterial origin. Vitamin K1 and K2 differ in the structure of their lipophilic side chain. Major menaquinones contain 4–10 repeating isoprenoid units (MK4 to MK10), produced from the microflora of the digestive tract and in the diet are present mainly in fermented foods [9]. The most relevant menaquinones in the diet are MK4 and MK7. Menaquinone-4 (MK4) is not a major constituent of bacterial production; instead, it is the product of tissue-specific conversion directly from dietary phylloquinone [10,11]. Menaquinone-7 (MK7) is the menaquinone of fermentative origin found in the Japanese traditional food natto, a soybean product fermented using *Bacillus subtilis natto.*

Despite its limited presence in other foods, MK7 has shown unique characteristics in terms of bioavailability and biological effects much superior to other components of the vitamin K family that have attracted remarkable interest in this molecule over the last decade [12,13]. In fact MK7 administered in the form of natto in equimolar amounts, compared to phylloquinone which is administered in the form of spinach, has a peak height difference of more than 10-fold and a half-life of 56 h compared to 7.5 h for phylloquinone [9] but also higher than MK4. Different chemical characteristics between K-1 and K-2, and among the latter a higher lipophilicity of MK7, appear to also influence tissue distribution and biological function of vitamin K. While all vitamin K molecule absorption appears to be initially associated with triglyceride rich lipoproteins (TRL), the longer chain menaquinones are also associated with low-density lipoproteins (LDL). The evidence has implications for MK7 transport to extrahepatic tissue, such as bone and vasculature, that are confirmed by a consistent amount of clinical evidence regarding the role of MK7 in bone and vascular health [14].

Due to its clinically confirmed biological efficacy, MK7 is an emerging molecule in the nutritional supplements market, where it is often used in association with minerals and vitamins that complete its role, in particular calcium salts and vitamin D3, which is a potent inducer of the major vit-K dependent protein in the bone, osteocalcin [15].

In the last decade, a large set of clinical studies highlighted the beneficial effects of menaquinone supplementation in the prevention of osteoporosis [16,17,18]. These studies employed natural MK7 produced by fermentation, the characteristics of which have been recently summarized in a US Pharmacopeia (USP) monograph, consisting of no less then (NLT) 96% and no more than (NMT) 101% of active all-*trans* MK7, along with NMT 2% of MK6, a characteristic marker of natural fermentation from *B. subtilis natto* that might be present in small amounts in natural products. Moreover, it is known that biologically inactive *cis* isomers can be formed as by-products in the chemical synthesis, as well as following geometrical isomerization, due to physical and chemical stress during technological processing or storage. In this respect, *cis* isomer content defined by the USP monograph in K2 should be NMT 2%.

Two recent publications [19,20] highlighted that the indications of the USP monograph showed important limitations in terms of purity profiling and are subjected to risk of overestimation since several *cis*-*trans* isomers cannot be distinguished from the all-*trans* active form with conventional chromatographic techniques. Using a combination of high-resolution mass spectrometry and quadrupole-time of flight (HRMS-Q-TOF) for the identification and charged aerosol/diode array detectors (CAD/DAD) for the quantification, Szterk et al. [19] observed that out of seven supplement formulations from the European and U.S. market, most had a lower content in active MK7 all-*trans* in association with relevant, in some cases major, content of inactive *cis*-*trans* isomers and other not identified impurities. Similarly, Jedynak et al. [20] have shown that the use of specific chromatographic columns and phases is able to detect potential contaminants in the raw materials, while insufficient separation is the main reason of overestimation of the results obtained by using the USP methodology when products are not highly purified. Notably, the same authors reported that MK7 was unstable in forced degradation experiments, in particular in alkaline conditions, suggesting that instability during storage or in formulation might be the cause of large variations in the content of MK7 in the studied dietary supplements, and they observed discrepancies between nominal and actual content. In particular, its use in formulation raised the question of potential instabilities of complex formulas, such as in combination with minerals or oxidising agents that could alter the stability of the formulation.

This evidence raises some serious concerns on the application of appropriate guidelines to guarantee quality and safety of dietary products. In fact, stability studies should include testing of those attributes that are susceptible to change during storage and formulation that are likely to influence quality, safety, and efficacy. In recent years, good manufacturing practices (GMP) have become mandatory also for dietary supplements [21], and guidelines originally intended for new drugs and active pharmaceutical ingredients have been borrowed from the International Conference for Harmonisation of Technical Requirements for Pharmaceuticals for Human Use (ICH) [22]. For this reason, it is very important to use standardized conditions and specific analytical methods to generate data also for food supplement applications, where quality attributes may sometimes not be fully defined in the product specifications or on the product label.

The aim of the present study was to investigate the stability of MK7 formulations from different origins and purity profiles, in order to clarify whether the latter may play a role in the stability of complex formulations.

In particular, two naturally fermented MK7 and a synthetic one, all commercially available, were used. All the active ingredients were independently formulated with minerals largely used on the market in combination with MK7, and stability was measured in standard and accelerated conditions. Finally, experimental observations were complemented by comparison of the actual/nominal content of MK7 in different formulations commercially available on the international markets, where the producers state on their labels the source of MK7 used to manufacture the finished products.

## 2. Results

### 2.1. Analysis of the Assay of Different MK7 Containing Powders

Three nominally identical MK7 powders containing 2000 ppm were preliminarily tested for their MK7 content. Powders were manufactured by different suppliers and with different production processes (i.e., PI–PIII were natural products of fermentation and PII was a synthetic product). Quantitative analysis reported in Table 1 shows that the content was in agreement with the nominal value, with a slight excess for PI. According to actual measurements, formulations of MK7 and mineral excipients were prepared at a concentration of 100 µg/g. 

The three product specifications did not show major differences, and the quality attributes are reported in Table 1. Since the products where compliant with the specifications, the mixtures with the excipients were prepared, consequently, for long-term and accelerated conditions stability testing.

### 2.2. Analysis of the Stability of MK7 and Mineral Containing Formulations

**Figure 2:**
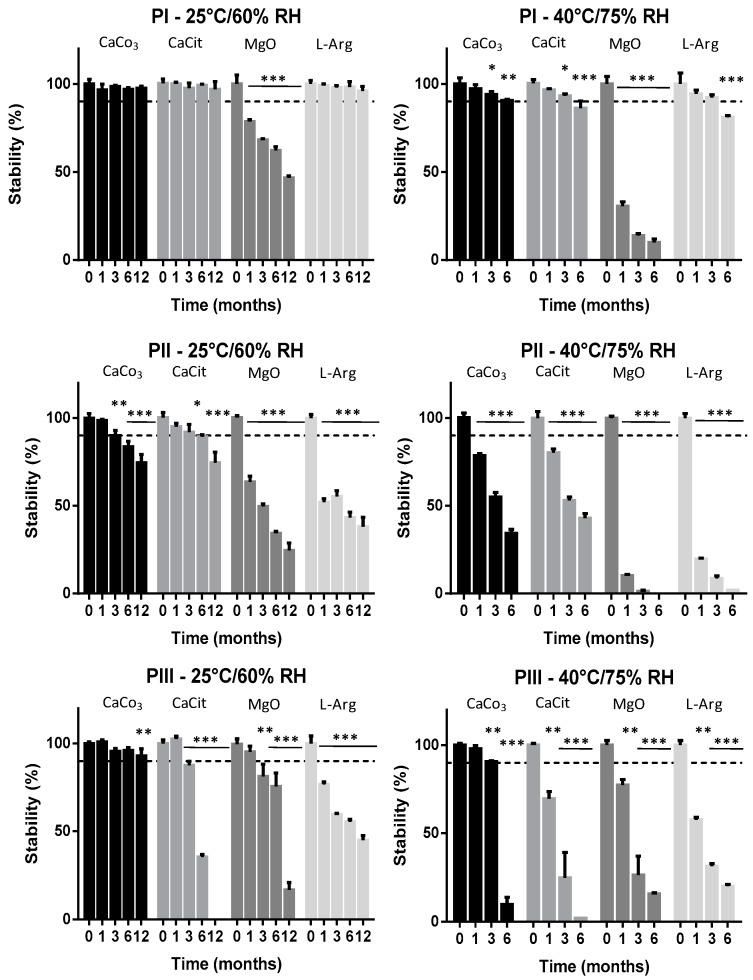
Stability of commercially available MK7 products, either from fermentation PI (Gnosis) and PIII (Sungen Bioscience) or from chemical synthesis PII (Kappa Biosciences), both in long term conditions (25 °C/60% RH; left panel) and accelerated conditions (40 °C/75% RH; right panel). At each experimental time, co-formulants are reported in sequence relative to different colors of bars: Formulation of MK7 with calcium citrate; calcium carbonate; arginine; and magnesium oxide. Data are reported as mean + SD. Experiments were conducted in triplicates. Significance of variation versus time zero values * *p* < 0.05; ** *p* < 0.01; *** *p* < 0.001.

Stability, summarized in Figure 2, was conducted up to twelve months at long-term (25 °C/RH 60%) and six months at accelerated conditions (40 °C/RH 75%) in duplicate samples analyzed independently. Analysis did show that while calcium salts, both citrate and carbonate formulations, were in general more stable, arginine (l-Arg) and magnesium oxide (MgO) might promote degradation of the sample, although product specific differences were observed with variations of the assay. In particular, while in the natural product PI, characterized by the highest content in all-*trans*, the stability was consistently higher for both calcium salts and arginine, with no statistical in stability over time, compared to the control up to twelve months in standard test conditions (Figure 2, left panel). In accelerated testing conditions, (Figure 2, right panel) stability of these compound was over 90% up to three months, although slight statistical differences with the control were detectable at the same time point (*p* < 0.05) and an even more significant degradation was observed in this experimental condition after six months, in particular, for Ca citrate and l-Arg (stability <90%; *p* < 0.001). On the contrary, magnesium oxide showed a marked and highly significant destabilizing effect, in particular, in the accelerated test conditions (*p* < 0.001).

In general, menaquinones, while showing a very high thermal stability, may be degraded following exposure to UV radiation and alkaline compounds [23]. It is very likely that magnesium oxide-associated instability is associated with alkalinisation, which appears to be remarkably effective in accelerated stress conditions in the presence of higher relative humidity (75%).

Interestingly, the destabilizing effect of the minerals was not identical in the presence of different MK7. In fact, the synthetic compound (PII) had already halved the MK7 content, in either the presence of L-Arg or MgO, after one month in standard conditions (*p* < 0.001), and, in general, all the tested compounds produced a significant decrease in stability, well below 90%, after 12 months in standard testing conditions (*p* > 0.001). In accelerated stress conditions, the stability profile of synthetic MK7 was worsened, showing an already highly significant decrease of MK7 in the presence of calcium salts after 1 month of storage (*p* < 0.001). Enhanced degradation was also observed for naturally fermented MK7 (PIII). In this case, degradation was also particularly evident for all minerals, in particular at 40 °C/75 RH, with the minor exception of calcium carbonate that remained stable for up to six months in standard testing conditions and up to three months in accelerated test conditions. In this case, all remaining formulation also significantly promoted degradation after one month of storage in both standard conditions, and this effect was clearly enhanced in accelerated conditions (*p* < 0.001).

### 2.3. Analysis of the Purity Profile of Different MK7 Containing Powders

Following the observed differences in stability behaviour in products with identical specifications, the purity profile of the vitamins was further analyzed using the methodology developed by Jedynak et al. [20], in order to detect any characteristics that may explain the differences in stability.

**Figure 3:**
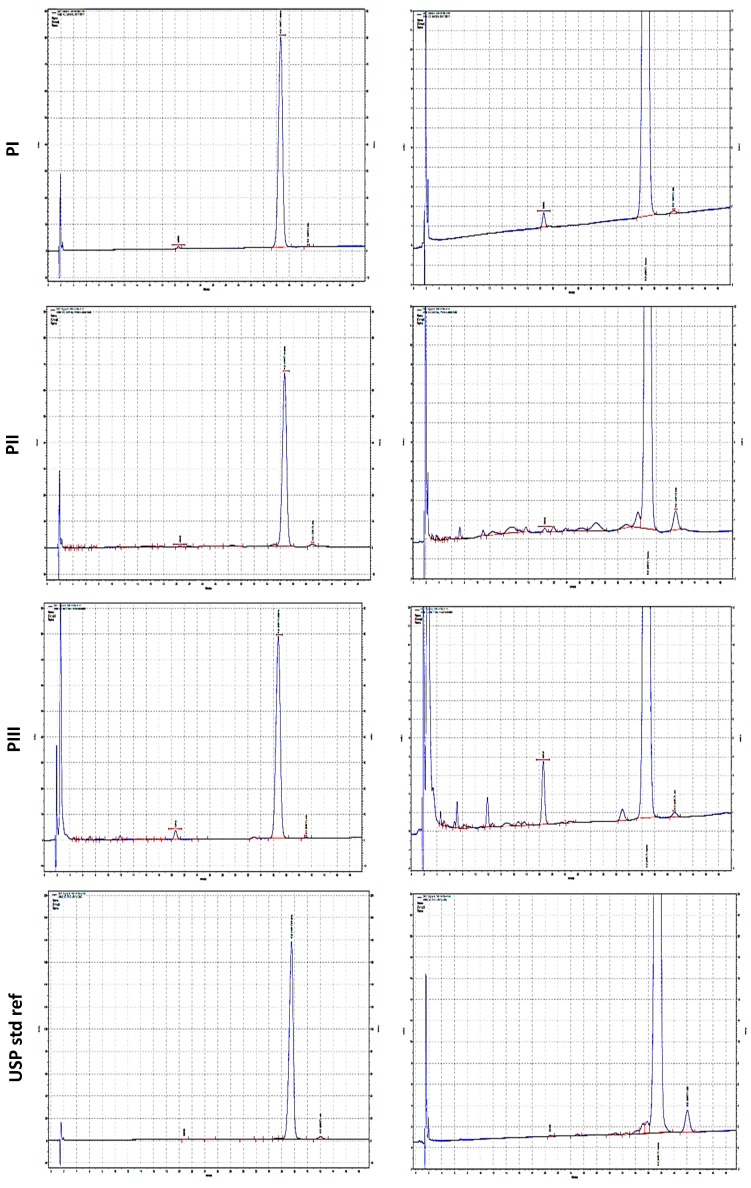
Chromatographic purity profile of commercially available MK7 products, either from fermentation PI (Gnosis) and PIII (Sungen Bioscience) or from chemical synthesis PII (Kappa Biosciences). The USP reference standard is also included. Right panel is a magnification of chromatographic profile highlighting the presence of impurities.

The results of the HPLC analyses showed very different chromatographic profiles, as reported in Figure 3. Analysis summarized in Table 1 shows that, while all producers comply with the general USP definition, they showed a very heterogeneous composition in impurities ranging qualitatively in the naturally fermented products from absence of undefined peaks (0% unknown components) in PI to 19 undefined peaks (3.52% unknown components) in PIII.

Synthetic MK7 PII showed the lowest purity profile, characterized by 23 species accounting for 5.67% of the unknown components.

### 2.4. Analysis of the Content and Purity Profile of MK7 Supplement Formulations on the Market

**Figure 4:**
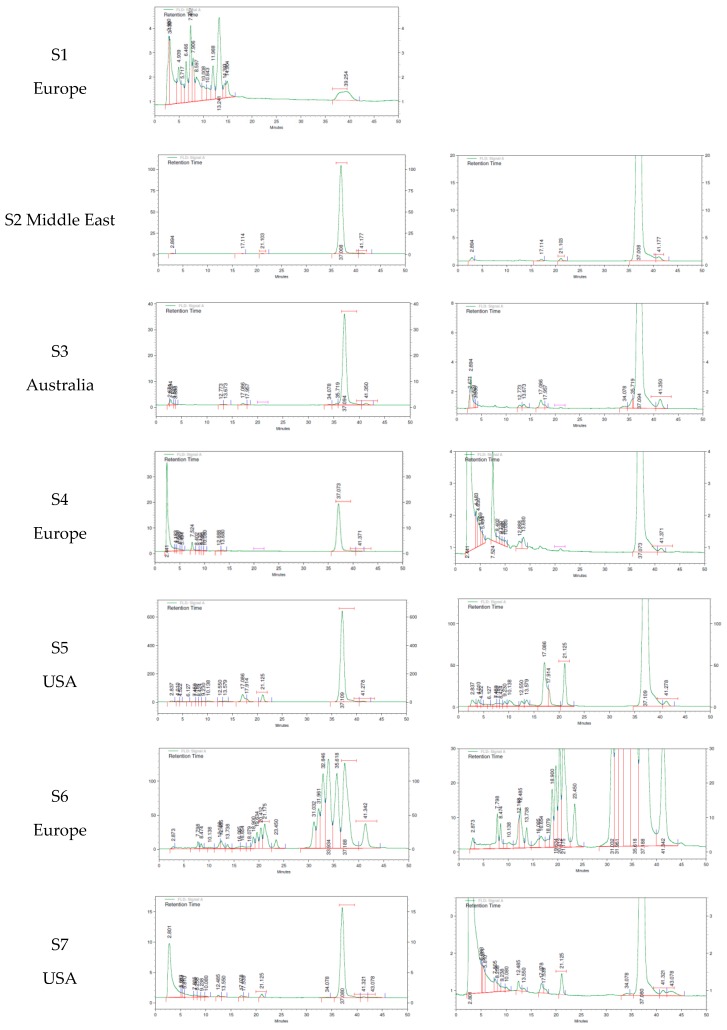
Purity profile of MK7 supplement formulations on the market. Supplier S1–S7 and their nationality is reported (S1–S7). The right panel is a magnification of the left panel, where analysis of minor components is more evident.

The MK7’s actual content and purity profile was estimated in seven different supplements, chosen among those available on the international market, mainly from suppliers declaring the source of menaquinone. Chromatograms are reported in Figure 4, and quantitative and purity data are summarized in Table 2. Analysis, conducted with the combined used of HPLC UV-visible for purity profile and fluorescent detection for sensitive menaquinone title definition, confirmed the presence of a variable number of undefined chromatographic peaks that ranged from 2 up to 19 compounds, in analogy to what was observed in the MK7 powder.

In particular, product S1 did report on its label the presence of MenaQ7® as an active ingredient, and the composition used did not include any mineral tested in the stability study. The assay was 55% of the claimed content in the label, and the shape of peak corresponding to Menaquinone 7 retention time was broad, which might suggest co-elution of multiple unresolved peaks. Moreover, the chromatographic profile shows 14 undefined peaks. The chromatographic profile was very poor, not allowing a precise identification of the origin of the active ingredient apart from its very low purity, and for this reason, a magnified chromatogram was not included as for the other products analyzed.

The S2 product contained VitaMK7® and calcium carbonate, which corresponded to one of the binary mixtures involved in the stability study. The content of the MK7 was in line with label claim, with a slight excess. The chromatographic profile was characterized by five peaks, with the major one corresponding to *trans* MK7, while two corresponded to *cis* isomer and MK6 (RT~21 min), which are known to be present in vitamins produced by fermentation, and the other two are undefined.

The S3 product contained K2Vital® and calcium citrate that corresponded to one of the binary mixtures involved in the stability study. Calcium citrate demonstrated an impact on the stability for one of the active ingredients tested; this could have justified the lower content found in this product, compared to the claimed value on the label (39%). The chromatographic profile was characterized by the presence of a total of 12 unidentified peaks.

The S4 product contained a complex mixture of different minerals, mainly as citrate salts. Vitamin MK7 used in the formulation was MenaQ7®. Similar considerations made for the S3 product also applied to this formulation. In particular, a lower content of MK7, compared to the claimed value on the label (36%), was likely associated to interactions with excipients present in the formulation. Regarding the purity profile, 13 unidentified peaks were noticeable; surprisingly, the unretained peak eluting at the solvent front showed intensity comparable to the vitamin peak.

The S5 product did not contain any of the minerals involved in the stability study, and the vitamin MK7 assay was in line with the amount claimed on the label, with a slight excess (116%). In this case, 13 unidentified impurities are visible in the purity profile, as well. Notably, the purity profile was different from S4, despite both formulations claiming the same source as the active ingredient (MenaQ7®). These differences could be related to product formulation.

Source of vitamins was not declared in S6, and the formulation contains none of the minerals tested in the stability study. The chromatographic suggested that it was formulated using a synthetic source. This was made evident by the presence of a high number of MK7-related peaks, which suggested the presence of multiple combinations of *cis* isomers and a second cluster of related peaks eluting earlier, that could have been related to a low purified intermediate having less isoprene groups. The MK7 content appeared to be in line with the label’s claimed values, but the purity profile showed that only 23% of the assay value could be associated with the active form of MK7.

No information about the vitamin source was available on the label for the S7 product, vitamin MK7 content was remarkably lower than the claimed values (5%), and none of the excipients used for the stability study were present in the formulation. There was a total of 15 unidentified peaks visible in the purity profile, including an unretained, unidentified peak that had an intensity comparable to the vitamin MK7 peak that was visible at the solvent front.

The presence of impurities was associated in four of the samples, with a notably decreased menaquinone content ranging from 5% (S7) to 55% (S1) of the nominal value. Only three of the tested supplements showed menaquinone content in agreement with the declared content, while the percentage of active all-*trans* form was above 97%, in agreement with USP guidelines, in only one supplement (S2).

There was a clear correspondence between the actual content and the purity profile, since the products with a lower content did show a large number of unknown impurities, as indicated in Table 2.

## 3. Discussion

In the present scenario of globalized manufacturing and distribution of dietary supplements, implementation of appropriate systems to ensure quality of the products is of paramount importance [21,24]. In fact, the absence of appropriate quality testing may lead to serious health consequences for consumers. This concept is consolidated in the development and manufacturing of drugs for medical use where GMP guidelines defined by ICH are mandatory. In the dietary supplement industry, GMP regulations were promulgated by the FDA almost a decade ago (21 CFR Part 111) [25], however, according to recent results of FDA GMP inspections [26], several manufacturers do not fully comply with cGMPs, often due to lack of specifications for ingredients and finished products. The consequences of the presence of unknown components in the finished products should not be underestimated, since they could influence the stability of the formulation and, most importantly, their safety is not guaranteed due to the absence of appropriate toxicity tests [27].

Following these considerations, this concept was applied to the present study for the evaluation of the purity profile and its relevance to formulation stability in different Menaquinone 7 (MK7) raw materials and formulations, either prepared in laboratory conditions or available on the market. Previous analysis of MK7 formulations available on the market have shown a high variability in the content of the active compound, which has been reported to be higher, lower, and even completely absent in certain formulas, as well as in the presence of inactive *cis* isomers with unknown toxicity profiles [19]. These inconsistencies are likely due to the aforementioned weaknesses in the regulatory requirements in the food supplement sector of the market, which are much less restrictive compared to pharmaceutical drug regulations.

In particular, combined comparative analysis of the stability and purity of different MK7 products reinforced the concept that materials which equally comply to minimal assay and composition characteristics, as detailed by the official USP quality standard for dietary ingredients, may be different in terms of their purity profiles due to the presence of *cis* isomers and other impurities.

USP sets standards for the identity, strength, quality, and purity of medicines, food ingredients, and dietary supplements manufactured, distributed, and consumed worldwide [28]. USP standards for drugs and dietary supplements are recognized in U.S. federal law and are enforceable by the FDA. However, although it is mandatory for drug product manufacturers to comply with USP standards, dietary supplement standards developed by USP are voluntary.

An official USP quality standard for a dietary ingredient, referred to as a monograph, sets forth the article’s name, definition, specifications (i.e., tests, test procedures, and acceptance criteria) and other requirements related to packaging, storage, and labeling. USP monographs include specifications for identity, assay, strength, composition, limits for contaminants, specific tests, and/or performance criteria (primarily for finished dosage units). In general, USP monographs for finished dosage forms do not refer to “purity” as a test in a monograph, but rather refer to an “Assay” or “Content” test procedure that is not suitable to measure the overall purity as referred to in FDA’s cGMPs when applied to an ingredient. The USP monograph for Menaquinone 7 sets maximum levels for Menaquinone 6 (assay) and for *cis*-Menaquinone 7 (isomeric purity) but does not require assessing of the overall purity nor sets a maximum level for unknown impurities. The test for strength in a USP monograph is used to measure the amount of a dietary ingredient per unit of measure in a dietary supplement, and it has the same meaning as in FDA’s cGMPs. The same applies to the MK7 USP monograph but, to our knowledge, none of the active ingredients that manufacturers give reference any test for measuring the overall purity in their current specifications.

Therefore, the present study also verified whether observed differences in the purity profile of the three active ingredients could contribute to different stability behaviour. In fact, data showed that different purity profiles corresponded to different stability profiles, with pure products generally showing the highest stability, with no statistical differences compared to the control up to 12 months of storage in standard conditions. Some of the tested excipients, such as magnesium oxide, promoted menaquinone degradation independently of the purity profile. This was likely due to a significant influence of this salt on the alkalinity of the formula. Nonetheless, the use of low purified ingredients has been shown to further trigger degradation once formulated with excipients.

This data, observed in experimental formulations, was confirmed in finished products available on the international market from different countries. In particular, it was shown that patterns of instability could be associated with types of MK7 characterized by different purity profiles. In particular, in the limited set of compounds tested, multiplicity of chromatographic impurities was frequently observed and consistently associated with lower content of the all-*trans* isomer (isomeric purity) or underestimation of the assay, despite the fact that an overage of the supplement formula is a common practice to minimize the risk of degradation and, consequently, lower assay of the formula. A limitation of the analysis of the finished products was due to the fact that we were not able to control previous storage and transport condition; nonetheless, conducted analysis constituted a random sample of marketed, not expired products representative of what was available on the market for the final consumer.

## 4. Material and Methods

### 4.1. Materials

Chemicals used in the study were Menaquinone 7: (PI) vitaMK7-vitamin K2 powder 2000 ppm–(Gnosis, Desio, Italy) lot 0001700112; (PII) K2VITAL–vitamin K2 powder 2000 ppm–(Kappa Bioscience, Oslo, Norway) lot PH-MCC2000-15-06;(PIII) NATTO K2–vitamin K2 powder 2000 ppm–(Sungen Bioscience Co., city, China) lot YK20160305a01; USP reference Standard lot R059X0; calcium citrate tribasic USP (ACEF SpA, Shantou, Italy); calcium carbonate for direct compression (ACEF SpA, Fiorenzuola D′Arda, Italy); arginine (Nutraceutica s.r.l, Monterenzio, Italy); magnesium oxide heavy Ph.Eur.(ACEF SpA); MENAQ7–vitamin K2 powder 2000 ppm (Nattopharma, Oslo, Norway) was not available at the time of the study, therefore, was not included in the evaluation.

Final products purchased on the market for comparison: (S1-Europe); (S2-Middle East); (S3-Australia); (S4-Europe); (S5–U.S.); (S6-Europe); (S7–U.S.). Complete description of formulation composition is reported in Table 3.

### 4.2. HPLC Assay of Vitamin MK7

The MK7 purity profile analysis was conducted according to the USP monograph [29] and ICH Q2 A/B guidelines [30]. MK7 solutions at a concentration of 32 µg/mL were prepared dissolving the vitamin in 1% tetrahydrofuran and bringing to volume with ethanol absolute; solutions were then filtered through the RC 0.45 µm filter. And then 10 µL of each solution were injected in an HPLC (Agilent 1100 series, Sant Clara, CA, USA) equipped with a Phenomenex Kintetex C18 100 mm × 4.6 mm × 2.6 µm at a flow rate 0.700 mL/min kept at 25 °C using as mobile phase a solution 97% methanol:3% water. Detection was conducted with a UV-268 nm, and the analysis time lasted 15 min. Specificity (Resolution MK6/MK7 = 1.77); linearity (r2 = 1.000); range 0.004–0.1 mg/mL; precision repeatability %RSD (*n* = 6) 0.52; intermediate precision %RSD (*n* = 12) 1.80; accuracy recovery = 102.3%; LOQ (S/N > 10) 0.002 μg/mL; LOD (S/N > 3) 0.001 μg/mL.

### 4.3. HPLC Analysis of Vitamin MK7 Purity Profile

The MK7 purity profile analysis was conducted according to Reference [20]. Briefly, MK7 solutions were prepared dissolving 5 mg USP MK7 RS or 1mg MK7 equivalent of powders representative of different production processes (I-III), in tetrahydrofuran and isopropanol (1:9); dissolved solutions were brought to a concentration of 0.1 mg/mL of MK7 using the same solvent and finally filtered through an RC 0.45 µm filter.

Eight microliters of diluted samples were directly injected in an HPLC (Agilent 1260 Infinity I) equipped with an Acclaim C30 250 mm × 2.1 mm × 3 µm at a flow rate of 0.400 mL/min kept at 15 °C using as mobile phase a solution of 98% methanol:2% water. Detection was conducted with a UV-248 nm, and the analysis time lasted 50 min.

Moreover, the method was also modified for the analysis of commercial formulas available on the market by inserting a post-chromatographic reducing column (Shiseido RC-10 30 mm × 4.0 mm) and a fluorescent detector (Agilent technologies G1321A FLD detector). Analysis of fluorescence (Ex 245 nm/Em 430 nm) allowed discrimination of menaquinone chromatographic peaks from those of other 254 light-absorbing substances, such as impurities or other ingredients.

### 4.4. Preparation of Formulations and Storage Conditions for Stability Study

Each MK7 active ingredient, once the actual content compared to the supplier certificate of analysis was verified, was mixed with appropriate amounts of mineral excipients in order to achieve a final concentration 100 µg/g of vitamin in the formulation. In order to avoid homogeneity issues during dispersion, a sample was prepared for each stability time point. In particular, three samples were made for each of the four formulations (36 samples that were further divided into two batches to be used for the standard and accelerated conditions). In total, 72 tubes were prepared and stored in dark polypropylene tubes in separate climatic rooms in the dark either in long term (36 samples at 25 °C/60% humidity) or accelerated stability conditions (36 samples at 40 °C/75% humidity) to be assessed after one, three, six, and twelve months of storing. At each time point, three samples were collected and analyzed. The study was performed in accordance with ICH [22], a guideline adopted by regulatory bodies of the European Union, Japan, and U.S. This guideline provides recommendations on stability testing protocols, including temperature, humidity, and study duration, and the purpose of stability testing is to provide evidence on how the quality of active ingredients varies with time under the influence of a variety of environmental factors, such as temperature, humidity, and light.

### 4.5. Statistical Methods

Quantitative experiments on the stability of MK7 in formulation have been performed in triplicate (*n* = 3) and data are reported as average of % stability compared to average time zero values and relative standard deviation. Normal distribution of the data was verified using the Shapiro Wilk test. Homoscedasticity was verified using the Bartlett test for comparing the variance of each set of data obtained from each formulation prepared at each time of sampling. Both assumptions were verified and, therefore, a parametric test statistical analysis of the data was performed. In particular, the significance of differences in means were calculated using 1-way ANOVA and Dunnett’s multiple comparison test for each time versus time zero values. Analysis was performed using Graphpad software, and *p* values ≤0.05 were considered statistically significant, with *p* values ≤0.01 and ≤0.001 considered highly significant.

## 5. Conclusions

In conclusion, although these data were preliminary and limited to the number of samples tested, the present study was in agreement with previous reports showing a general instability issue in MK7 formulation and suggested that purity profile aspects should be taken into account while choosing MK7 for formulation purposes in order to guarantee efficacy, safety, and stability of the product.

In fact, the *trans* form is the only active form of menaquinone able to act as a cofactor for carboxylation; on the contrary, *cis* isomers are associated with lack of activity, unknown toxicity profile, and may contribute to instability.

The current USP monograph for MK7 can serve as a useful resource to help dietary ingredients manufacturers comply with cGMP’s when setting quality specifications. Manufacturers can benchmark their tests, analytical procedures, and acceptance criteria against those in USP monograph when setting a specification for MK7, but an update of the current procedure may be required since there are no explicit requirements to characterize all the impurities contained in the products, and no one manufacturer seems to indicate that they follow this monograph in setting their product specifications.

ICH Quality Guidelines can also offer manufacturers a scientifically valid mean of supporting compliance with the specification requirements described in the cGMP, application of an approach in line with principles expressed in ICH Q3A for the classification of impurities in by-products, intermediates, and degradation products, and can help enhance the quality and safety of the dietary supplement marketplace and protect public health.

Appropriate analytical methodologies and updated USP guidelines should also consider these aspects in the characterization of the molecule.

## Figures and Tables

**Table 1:** Ingredients assay and specifications and purity profile.

Test Product	PI	PII	PIII
*Producer*	*Gnosis*	*Kappa Bioscience*	*Sungen*
Appearance	Light yellowish powder	White to light yellow fine powder	Yellowish powder
Loss on drying	≤5%	<5%	<5%
Total heavy metals	≤10 ppm	≤10 ppm	≤10 ppm
Total aerobic microbial count	≤10^3^ CFU/g	≤10^3^ CFU/g	≤10^3^ CFU/g
Total combined yeasts & molds	≤10^2^ CFU/g	≤10^2^ CFU/g	≤10^2^ CFU/g
Declared quantity MK7	2000 ppm	2000 ppm	2000 ppm
Actual quantity MK7	2066 ppm	1911 ppm	2264 ppm
% actual/declared	103%	96%	113%
All-*trans*-MK7 (area%) (US Pharmacopeia (USP) 97.4)	99.3	92.7	93.7
*cis*-MK7 (area%) (USP 1.2)	0.2	1.4	0.3
MK6 (area%) (USP 0.03)	0.6	0.2	2.5
^#^ undefined chromatographic peaks (USP 6)	0	23	19

**Table 2:** Products assay and purity profile.

Suppl.	Provenience	Active Ingredient Source	All *trans*-MK7 (Area%)	*Cis*-MK7 (Area%)	MK6 (Area%)	^#^ Underfined Chromatographic Peaks	Label Claim (μg/unit)	Actual Conten (μg/unit)	%
S1	Europe	MenaQ7	7.4	-	-	14	100	55	55
S2	Middle East	VitaMK-7	97.8	0.8	0.3	2	90	105	117
S3	Australia	K2 Vital	90.6	1.7	-	12	30	12	39
S4	Europe	Mena Q7	48.9	0.3	-	13	25.7	9.3	36
S5	USA	Mena Q7	81.8	0.8	4.2	13	100	116	116
S6	Europe	Not declared	23.4	5.3	4.1	19	200	212	106
S7	USA	Not declared	52.7	0.6	1.2	15	100	5	5

**Table 3:** Product composition (from product labels).

Finished Product Supplier Code	S1	S2	S3	S4	S5	S6	S7
Composition	Fish oil	Ca carbonate	Ca citrate	Ca citrate	Microcrystalline Cellulose	VegetableGelatin	Ca hydrogenphosphate
	Gelatin animal origin			Mg citrate	Modified Cellulose	Microcrystalline Cellulose	Dextrin
	Glycerol			Fe citrate	Si dioxide		Hydroxypropylmethyl cellulose
	Orange flavor			Zn citrate	Glycerolmonostearate		Magnesiumstearate
	Tocopherol			Mn sulphate	AscorbylPalmitate		Si dioxide
	Calcipherol			Cu citrate	Rosemary exctract		Na carboxymethyl cellulose
				K iodide			
	Docosahexaenoic acid			Cr picolinate			
	Eicosapentaenoic acid			Na selenate			
				Na molibdate			
				Vitamin A Vitamin C Vitamin D3 Vitamin E Vitamin B1 Vitamin B2 Niacin Pantothenic acid Vitamin B6 Biotin Folic acid Vitamin B12			
